# Is it the “public” health system? The VOICE model for inclusiveness in universal (national) health systems - lessons from COVID-19

**DOI:** 10.3389/fpubh.2023.1243943

**Published:** 2023-12-13

**Authors:** Gillie Gabay

**Affiliations:** Achva Academic College, Shikmim, Israel

**Keywords:** inclusiveness, health policies, religious minorities' communities, lessons from COVID-19, equity, public health

## 1 Introduction

Since its inception, Israel has provided universal public health care for all its citizens via four not-for-profit health maintenance organizations (HMOs). Definitions of public health abound, including the organized response by society to protect and promote health, to prevent injury, illness, and disability, and to “provide the necessary conditions for a population to be healthy (1). Universal or national health systems view the right to health as a human right (2). Based on that view, the Israel National Health Insurance Law (3) made health insurance mandatory, stipulating that every resident, irrespective of age, religion, ethnicity, or socio-economic status, is entitled to an exceptionally broad basket of health services (4). The implementation of the law has indeed increased equality though inequalities still exist (5–9). Most of the inequality in health policies and services relates to religious minorities such as the Ultra-Orthodox Jews and Muslim Arabs (10, 11).

The inequality in a universal system is a major concern of policy makers and health authorities who make continuous efforts to reduce inequalities and shape culturally sensitive policies for planning and delivery of care (4, 8, 12, 13). Sensitive policies include social participation of all members of society, which albeit being a complex undertaking in practice, is acknowledged by the WHO as an important means for policymakers to develop responsive health policies with a higher likelihood of being implemented (14). A universal health system, by definition, needs to ensure the right to equitable physical and mental health so that rights are not infringed, and policies don't negatively weigh outcomes on both marginalized groups and society (2). Inequity in the Israeli universal system due to cultural differences has been studied (4, 15, 16). Research on policies and inequalities during COVID-19 and their effect on wellbeing, however, remains remarkably absent (17). The pandemic generated an opportunity to explore inequity underlying health policies that were applied to the health crisis as a response by policymakers and authorities. The pandemic warrants an exploration of the policies as means of reducing inequities by inclusiveness (16, 17).

The concept of inclusive health focuses on good health and well-being for everyone. This concept resonates with a rights-based approach to health including political, social, economic, scientific, and cultural actions that are geared toward advancing good health and well-being for all (18). This study seeks to enhance the understanding of the linkage between health policies during a health crisis, inclusiveness, and inequities, using COVID-19 policies in Israel as a case example. The topic of this study falls within the second group of the basic framework for One Health research which is captured in The World 2050 Initiative (TWI) (19). This author argues for the need to form inclusive health responses, not only in routine but also during public health emergencies for public health promotion. Insights from this study may inform policy makers and health authorities of universal systems and direct interventions for reducing inequities and ensuring inclusiveness.

### 1.1 Religious minorities in Israel

There are seven religious minorities in Israel comprising 37.5% of the population. In Israel, the ultra-Orthodox Jewish community, and the Arab population (Muslim and Christian) are the most prominent and well-defined minority groups (20, 21). According to the Israeli Central Bureau of Statistics 2018, there are three population groups: Jews, who constitute 75% of the population; Arab Israelis, who account for 21% of the population, and others, 4% of the population which include non-Arab Christians, Buddhists, Hindus, Samaritans, and Bahá'ís. In 2015, the Arab Israeli population reached 1.8 M and is comprised of 84% Muslims, 8% Druze, and 8% Christians. Bedouins account for 16% of the Muslim population (22). These religious minorities are collectivist, and the identity of the group transcends that of the individual (23). The ultra-Orthodox and Muslim minorities have large families, live in close-knit communities, have death rituals that distinguish them from other marginalized groups, have a complicated relationship with the government and their behavior is guided by their spiritual leaders (24).

### 1.2 Religious minorities in Israel during COVID

Compliance with physical distancing across religious minorities has been shown to be relatively poorer compared to the majority group (23, 25, 26). The Jewish ultra-Orthodox minority comprises 12.6 of the population but 40–60% of all coronavirus patients were Jewish ultra-Orthodox; similarly, the Arab population comprises 21% of the population but 33% of all coronavirus patients were Arab (27). The social representation theory stresses the need to design culturally adapted messages to reflect the shared reality of each marginalized group (28). Throughout COVID-19, however, although risk perception has cultural roots, messages calling for physical distancing were not culturally adapted, leading to poor compliance with guidelines (29, 30).

### 1.3 Health policies during the pandemic and inclusiveness in Israel

The COVID-19 pandemic, a public health emergency, affected many countries worldwide and necessitated measures to contain the virus. Like other countries, Israel implemented measures of preservation of hygiene, mandatory mask wearing, and maintaining physical distancing (27, 31, 32). Physical distancing refers to maintaining physical separation to reduce close contact between people (33). Practices of physical distancing included self-isolation, quarantine, preventing assemblies of people in community settings, and closures of schools, gyms, bars, and restaurants (34, 35). Physical distancing remained the primary intervention throughout the five waves of the pandemic (36, 37). Health authorities invested efforts to implement the physical distancing policy by education, persuasion, legislation, incentives, and coercion, which offended members of certain religious minorities (33, 38). These measures to contain the virus prevented communal traditions, including religious practices, death rituals, and funerals (34, 35). Coercion infringed human rights, and lead to higher stress, distress, family conflicts, and loneliness (39).

Although collective religious practices foster connectedness and resilience, there were no platforms to foster spirituality throughout the adversity faced by minorities (40–42). Furthermore, minority members claimed that activities in synagogues and mosques, which are critical for spirituality, for social support of bereaved, for family conflict resolution, for marriages, divorces, funerals, and for individual counseling, were banned under the guidelines, without any discussion with spiritual leaders about safe alternatives that had been applied in previous pandemics (41). Other public places (i.e., parks, bars) were allowed, from the third wave of the pandemic, to open whereas places of worship remained closed, leading to disappointment and anger at health authorities and the disengagement of minority members and their leadership from the fight against COVID-19.

Due to their poor compliance with guidelines, members of religious minorities, who had to cope with linguistic, employment-related, and socioeconomic challenges, became more vulnerable as it was harder for them to understand and apply public health measures (43). Members of religious minorities faced heightened discrimination by the majority group (23). The involvement of the police to enforce the health guidelines created fear and a sense of humiliation (41). Reports in the mass media depicted members of religious minorities as lacking respect for the physical distancing measures and jeopardizing public health (44).

Media reports kept exposing the lack of compliance of the Jewish ultra-Orthodox population with the law mandating hospitalization in public hospitals in cases of severe COVID-19. To avoid having their followers die in solitude, in overflowing, understaffed hospitals, the leadership of the ultra-Orthodox established underground hospitals which were an innovative implementation of patient-centered care in a health crisis (45). Several months later, the health authorities themselves adapted the policy enabling hospitalization in the community of patients with moderate COVID-19.

Moreover, the disempowerment of the religious minority spiritual leaders responsible for the sustainability of their communities during the five waves of the pandemic caused a breach of trust between these communities and the health authorities and inhibited the support of religious leaders for public health measures (41). COVID-19 guidelines inhibited the practice of death rituals and created multi-level clashes between values and beliefs and guidelines, inhibiting effective processing of grief and effective functioning following loss. Muslims, in particular, experienced disenfranchised grief at the individual and community levels, jeopardizing wellbeing and leading to distrust of authorities and policymakers, deeper polarization in the society, lower utilization of health services, poor mental health, and worsening of chronic illnesses (41).

### 1.4 Actions of health authorities

Core activities of public health include community education, outbreak investigation and communicable disease control, risk factor and disease surveillance, screening, development and implementation of public health interventions, evaluation, and research. Since the 1970s, public health has been emphasizing the partnership approach, which aims at community engagement, health promotion, and inter-sectoral partnerships (1). The partnership and collaboration approach throughout COVID-19 in Israel, however, was deficient.

Health authorities and policy makers were perceived by members and leaders of religious minorities as failing to recognize the loss of community; as devaluing the spiritual leaders of religious minorities who are responsible for the continuation of community and excluding them from decision making; as unaware of potential adaptations to religious values and beliefs that were possible in previous pandemics; failing to provide resources to help community members plan for practical needs after death; failing to improve wellbeing and resilience through grief counseling and self-care for elders during this challenging time; failing to create and make accessible communication platforms that alleviate anxiety and process collective loss in order to restore social identity (24, 41, 41, 45, 46). These experiences resulted in distrust, disappointment, and anger at health authorities, rejection of the vaccine, poor compliance with guidelines, ineffective grief, deeper polarization from the majority population, and poor utilization of health services.

## 2 Discussion

This study explored the inclusiveness of health policies during the pandemic in the Israeli universal health system. The pandemic revealed weaknesses and blind spots in responding to it as a universal health system should. Although reducing inequities by community engagement has been promoted as a key element of epidemic responses, a collaborative approach with minority leaders was found to be lacking (24, 41, 46–48). Responses to the pandemic exacerbated inequalities that already existed; marginalized underrepresented groups were reported to be left behind and discriminated against partly due to COVID-19 policy responses (2, 24, 41). Highlighting the system's blind spots, the COVID-19 pandemic demonstrated how important it is to ensure an inclusive health approach to health emergencies.

Inclusive responses to public health emergencies are a tenet of public universal health systems (18). Since it was important to balance risks to public safety the insensitivity toward minorities, may have been understandable through the second wave, but from the third wave onward, authorities could have engaged in ongoing negotiations with leaders of religious minorities as the crisis evolved. Lack of inclusion was detrimental to achieving a commitment from minority populations to respect the control measures advocated by public health authorities (49). As in other countries, health authorities in Israel may have underestimated the capacity of the leadership and of members of these religious minorities to become active in designing the response to the pandemic (50). In the context of the COVID-19 pandemic, health responses failed to be empowering and to enhance wellbeing. Voices of minorities were excluded from social discourse and thereby from policy making. Although a public universal system is expected to attend and serve all citizens, this author demonstrated that religious minorities experienced non-inclusive public health responses throughout COVID-19 (2, 24, 41, 44).

Failing to recognize and address the needs of every marginalized group in the population based on the right to health led to distrust and translated into less compliance, as was demonstrated in Israel by 12% rejection of the first two vaccines, mostly by members of marginalized groups and by 48% rejection of the subsequent boosters (44, 50). If leaders of a marginalized group are excluded from decision making on policy and guidelines, the efficacy of a pandemic response may be severely undermined (49, 51). Exclusionary policies may have a long-term effect on utilization of health services long after the pandemic by neglecting alternative understandings that challenge dominant constructions of health and healthcare (40). Such neglect weakens the capacity of participatory action to promote transformative change through dialogical orientation (51, 52). Moreover, it may produce or exacerbate health inequities, as policies and services become increasingly adapted to the demands of vocal majorities (23).

Participation of leaders of marginalized groups in decision-making foster culturally adapted policies which make more responsive policies, and, consequently, healthier populations (53). Initiatives that focus on community empowerment are increasingly prominent in public health policy. However, while participation and inclusion are necessary conditions for empowerment, attention to the breadth of inclusion and to the extent to which it is experienced as empowering was insufficient (54). Several recommendations are proposed to promote inclusiveness in responses of universal systems to a health crisis.

### 2.1 Practice implications for policy makers and health authorities

Health authorities have a role in promoting the substantive inclusion of marginalized groups in healthcare decision-making (51). Health authorities must be strategic and proactive in reaching out to specific groups, to identify and address their needs, to disseminate transparent and accurate public health information, and shape actionable options to enhance public trust in health authorities and policymakers (49). Actively listening to leaders of religious minorities can facilitate collaborations with the communities which will reduce tensions through the process of reclaiming trust in authorities and policy makers. A collaborative approach may lead to open conversations that elucidate the challenges that religious minorities experience and the effects on equity and public health principles, especially in universal health systems.

There are implications for policy makers and authorities at three levels: Leadership, community levels, and collaborators. At the leadership level, to establish trust of leaders and their communities, in authorities, health authorities must validate the leadership of minorities by approaching it, initiating dialogues, providing transparent empirical data, and understanding disparities in approach and objections. Authorities and policymakers should avoid top-down imposition of solutions using a one-size-fits-all model (55). At the community level, authorities are called upon to understand the sources of objection and distrust and use culturally appropriate channels to communicate. Regarding collaborators, this author proposes to engage clinicians from religious minorities and collaborate with them to initiate community-based efforts to understand their needs, concerns, and possible solutions.

**Figure 1:**
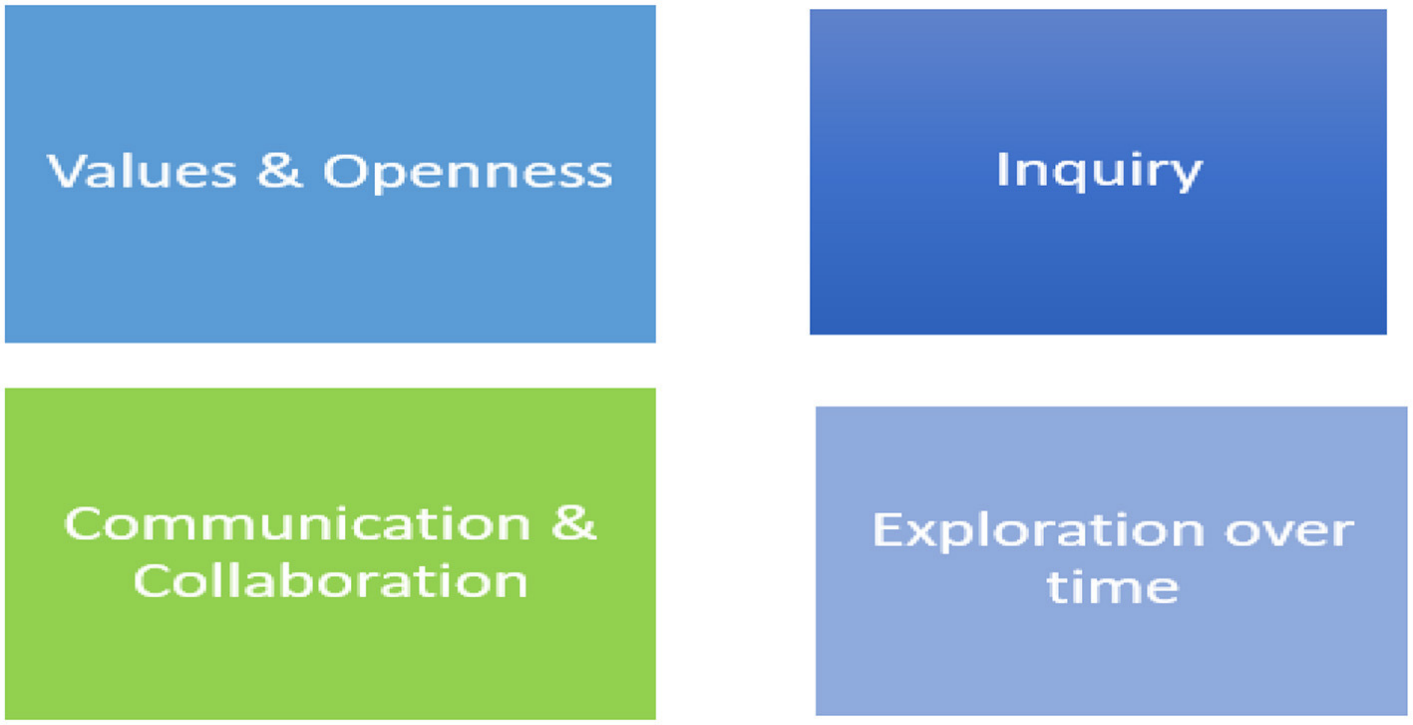
The VOICE model for inclusiveness in universal health systems managerial implications.

To succeed in promoting inclusiveness, authorities, and policy makers should apply the VOICE model in several steps. First explore their *Values* regarding inclusiveness in universal health systems. Second, be O*pen*: the knowledge does not lie only with policymakers. Reflect and ask what we learned during COVID-19. Although there is a universal health system that aspires to eliminate inequities, state policies in practice reduced inclusiveness and enhanced perceived and actual inequities between the majority population and religious minorities. Third, *Inquire* about needs, concerns, objections of minorities. Fourth, *Communicate and Collaborate* to develop possible culturally adapted alternatives to the policies to preserve public health on the one hand and to respond in a way that includes religious minorities, on the other. Fifth*, Explore* alternatives over time. Figure 1 presents the VOICE model.

Looking forward, health authorities may intervene to ensure inclusiveness in health services for all members of society. Ensuring inclusive health responses is important in addressing health inequities in the short and long term (56). This author proposes six interventions to foster inclusiveness: (a) Create future inclusiveness through leadership education making sure that the health authority boards that make policy decisions reflect the diversity of the population. (b) Train the next generations of leaders by inclusive communication, public engagement, involved networks, as well as recruiting agents from each minority and setting up advisory groups. With time these measures will help the leaders of religious minorities to gain influence on shaping policies and help reclaim trust in policy makers health authorities. (c) Employ measures to prevent the infringement of civil rights of minorities and assess it as an important measure for health quality (d) Reward practices of inclusiveness to direct conduct toward this goal. (e) Critically appraise practice by evaluating and measuring trust, polarization, and utilization of health services.

## 3 Conclusions

Inclusive, dynamic, multi-stakeholder, responses of health systems remain critical in the context of health emergencies. Responses of public health authorities and health systems are to be more strategic, proactive, and inclusive, reaching out to all minorities and addressing their specific needs, values, and beliefs. Such an inclusive health approach may foster solidarity and health equity, perhaps leading to more effective responses of religious minorities to public health interventions in both emergencies and routine. Shaping strategies to target diverse multi-stakeholders is a first step to engage everyone in society, reduce discrimination, health inequity, and health deterioration. A commitment to an inclusive system implies that activities and responses will be sensitive to all, so no minority is left behind. This maintains the commitment of health systems to be universal health systems, for everyone, including in emergencies. As the COVID-19 pandemic, an unprecedented humanitarian crisis, continues to threaten and impact public health systems around the world, disrupting the well-being of people, inclusiveness in public health universal systems, cannot only exist in declarations. Inclusiveness cannot be overlooked. Policies should link policymakers and public health authorities with members and leaders of religious minorities to better respond to the needs of all.

## Author contributions

The author confirms being the sole contributor of this work and has approved it for publication.
